# Age-Related Decline in the Variation of Dynamic Functional Connectivity: A Resting State Analysis

**DOI:** 10.3389/fnagi.2017.00203

**Published:** 2017-06-30

**Authors:** Yuanyuan Chen, Weiwei Wang, Xin Zhao, Miao Sha, Ya’nan Liu, Xiong Zhang, Jianguo Ma, Hongyan Ni, Dong Ming

**Affiliations:** ^1^College of Microelectronics, Tianjin UniversityTianjin, China; ^2^Tianjin International Joint Research Center for Neural Engineering, Academy of Medical Engineering and Translational Medicine, Tianjin UniversityTianjin, China; ^3^Department of Biomedical Engineering, College of Precision Instruments and Optoelectronics Engineering, Tianjin UniversityTianjin, China; ^4^Department of Radiology, Tianjin First Center HospitalTianjin, China

**Keywords:** aging, dynamic functional connectivity, functional connectivity variation, functional connectivity fluctuation frequency, resting-stated fMRI

## Abstract

Normal aging is typically characterized by abnormal resting-state functional connectivity (FC), including decreasing connectivity within networks and increasing connectivity between networks, under the assumption that the FC over the scan time was stationary. In fact, the resting-state FC has been shown in recent years to vary over time even within minutes, thus showing the great potential of intrinsic interactions and organization of the brain. In this article, we assumed that the dynamic FC consisted of an intrinsic dynamic balance in the resting brain and was altered with increasing age. Two groups of individuals (*N* = 36, ages 20–25 for the young group; *N* = 32, ages 60–85 for the senior group) were recruited from the public data of the Nathan Kline Institute. Phase randomization was first used to examine the reliability of the dynamic FC. Next, the variation in the dynamic FC and the energy ratio of the dynamic FC fluctuations within a higher frequency band were calculated and further checked for differences between groups by non-parametric permutation tests. The results robustly showed modularization of the dynamic FC variation, which declined with aging; moreover, the FC variation of the inter-network connections, which mainly consisted of the frontal-parietal network-associated and occipital-associated connections, decreased. In addition, a higher energy ratio in the higher FC fluctuation frequency band was observed in the senior group, which indicated the frequency interactions in the FC fluctuations. These results highly supported the basis of abnormality and compensation in the aging brain and might provide new insights into both aging and relevant compensatory mechanisms.

## Introduction

Normal aging in the human brain refers to degradation phenomena that occur in brain structures, brain function and brain morphology with increasing age, indicating that a certain degree of senior brain dysfunction will occur (Hedden and Gabrieli, 2004; Fjell et al., 2014). Considering the increasing size of the aging population, the incidences of diseases that are highly associated with age, such as Alzheimer’s (Kern and Behl, 2009; Mosher and Wyss-Coray, 2014) and Parkinson’s (Xu et al., 2012; Reeve et al., 2014), are also increasing. Until now, the mechanism of aging has remained unclear, and further investigation of brain aging could greatly help in managing problems associated with both aging and disease.

Functional connectivity (FC) based measures of the resting state functional magnetic resonance imaging (rs-fMRI), which reflect the coherence between temporal fluctuations across brain regions, are organized into distinct systems or networks (Damoiseaux et al., 2008; Zuo et al., 2010). Most studies of FC have focused on the decline of specific functional systems, such as the default mode network (DMN; Raichle et al., 2001; Damoiseaux et al., 2008; Wang et al., 2010; Ferreira and Busatto, 2013), or have focussed on other specific brain networks or regions, such as the language system (Zou et al., 2012), subcortical regions (Yi et al., 2015) or the motor system (Coynel et al., 2010; De Vico Fallani et al., 2013). Increasing evidence has shown that the decline in cognitive function associated with aging is related to changes in communication between different brain regions and subsystems (Andrews-Hanna et al., 2007; Sambataro et al., 2010), even in the resting state (Shehzad et al., 2009; Meindl et al., 2010; Guo et al., 2012; Zuo et al., 2013). Despite this progress, how brain systems cooperate to handle aging-associated declines remains unclear, especially considering the averaging of complex spatiotemporal phenomena during a period of time (Hutchison et al., 2013a).

Traditionally, functional connectivities derived from fMRI data are computed using signals across the entire scan time; it is assumed that the functional connectivities among the brain regions are static during the duration of the resting time (Handwerker et al., 2012; Zuo et al., 2013). However, recent work has shown that FC is temporally dynamic (Chang and Glover, 2010; Calhoun et al., 2014) even at rest. This dynamic FC, which varies over a timeframe of seconds, could be highly related to unconstrained mental activity during the resting state (Hutchison et al., 2013a; Allen et al., 2014; Zalesky and Breakspear, 2015) and even under anesthesia (Hutchison et al., 2013b). A widely applied method for analyzing temporal dynamics is the sliding window correlation method (Sakolu et al., 2010; Hutchison et al., 2013a; Di and Biswal, 2015). A series of FC matrices was obtained using this method, which showed the time-varying connectivity network. Thus, researchers have started to perform dynamic FC investigations of mild cognition impairment (Wee et al., 2016), epilepsy (Liu et al., 2016), schizophrenia (Rashid et al., 2014; Du et al., 2016), major depressive disorder (Demirta et al., 2016) and normal development (Sakolu et al., 2010; Rashid et al., 2014; Qin et al., 2015).

The convergent results of previous studies have suggested that the dynamic resting state FC is highly intrinsic and physiologically relevant. Several studies have reported that the FC states revealed by changes in connectivity over the course of the scan can be sensitive to changes related to neurological disorders (Sakolu et al., 2010; Li et al., 2014; Leonardi and Van De Ville, 2015; Ou et al., 2015; Shakil et al., 2016). Increasing efforts have been directed toward using functional microstates and their transmissions to depict the working mechanisms of the brain (Allen et al., 2014; Shakil et al., 2016). Microstate transmissions could be the bases of integration and segregation between different brain networks or cognitive resources (Hansen et al., 2015; Yu et al., 2015; Shakil et al., 2016). Previous work has shown that aging impacts not only within-network connectivity but also the integration and segregation of different brain networks (Ferreira and Busatto, 2013). Advancing age induces increased reorganization to establish compensatory mechanisms or plasticity that counteract the aging process (Meunier et al., 2014; Sala-Llonch et al., 2015; Sugiura, 2016). Segregation and integration are the bases of reorganization of the brain connectivity network. The investigation of dynamic FC in the resting state may provide new insights into communication between various cognitive resource pools in the aging brain. The derived patterns of temporal variation in FC thus reflect the interactions of the brain functional networks and are therefore expected to facilitate our understanding of the mechanisms that underlie mental diseases.

We expected that the fluctuations of resting FC comprise a dynamic balance that maintains the intrinsic connectivity patterns in the brain. The dynamic balance of FC allows us to capture the interactions between all of the subsystems and the basic states of brain connectivity. Since the FC levels and patterns are age-related, this dynamic balance and the serial connectivity networks must also change with increasing age. A previous study (Leonardi and Van De Ville, 2015) suggested that the spontaneous fluctuations in the FC have frequency dependence and result from the interactions of various frequency components associated with neural activities. Limited resources and decreased processing speed can indicate performance during aging; thus, we hypothesized that the dynamic FC can provide clues for the capacity and efficiency of the connectivity states that transfer and present aging features. This article focuses on revealing the effects of aging on the time-varying FC of the brain in the resting state. Using the sliding window correlation method, the resting state fMRI data from two groups of young and senior healthy individuals were processed to construct dynamic FC matrices. We expected that the variation and the frequency spectrum of the FC fluctuations were the important bases of the dynamic balance and were highly related to aging.

## Materials and Methods

### Participants and fMRI Data Acquisition

All resting-state fMRI data used in this study were obtained from the NKI-Rockland Sample (NKI-RS^1^), which is provided by the Nathan Kline Institute (NKI, Orangeburg, NY, USA) and is available online in a public database. To study the changes in the dynamic characteristics of FC that resulted from normal brain aging, we collected fMRI data from 68 healthy subjects who were organized into two groups: 36 young subjects were assigned to one group (mean age, 28.1 years; range, 20–35 years; 24 male), and 32 senior subjects were assigned to the other group (mean age, 70.6 years; range, 60–85 years; 15 male). According to the demographic information provided by the NKI-RS data set, there was a remarkable difference in the age of the participants, but no significant differences in either gender or hand dominance between the two groups.

Resting-state fMRI data were collected using an echo-planar imaging (EPI) sequence on a 3.0 T SIMENS Trio scanner. The scanning parameter settings were as follows: TR/TE = 2500/30 ms, flip angle (FA) = 80°, field of view (FOV) = 216 × 216 mm^2^, voxel size = 3 × 3 × 3 mm^3^, number of slices = 38, scan time = 650 s time points = 260. During the data acquisition, the subjects were instructed to keep their eyes closed and to stay awake. High-resolution T1-weighted images were also acquired using the magnetization-prepared rapid gradient echo (MPRAGE) sequence. The acquisition parameter settings were as follows: TR/TE = 2500/3.5 ms, FA = 8°, FOV = 256 × 256 mm^2^, voxel size = 1 × 1 × 1 mm^3^, slice = 192.

### Data Preprocessing

Functional images were preprocessed using the Connectome Computation System (CCS^2^). The CCS designed by Zuo et al. (2013) provides a computational platform for multimodal neuroimaging brain connectomics computations by integrating the functionalities of AFNI, FSL and FreeSurfer (Zuo et al., 2013; Betzel et al., 2014; Cao et al., 2014). The functional preprocessing included the following: the first ten functional volumes were discarded to allow for signal equilibration; slice timing was corrected using the middle slice as the reference frame; 3D geometrical displacement was used to correct for head motion; and 4D global mean-based intensity correction was performed. In addition, the Friston-24 model was used to remove micro-level motion artifacts (Friston et al., 1996) and nuisance regressors; for instance, the individual white matter and the cerebrospinal fluid (CSF) mean signals were regressed out. The functional data were also temporal band-pass filtered (0.01–0.1 Hz) and detrended (both linear and quadratic trends). Finally, spatial smoothing was performed with a Gaussian filter kernel (FWHM = 6 mm). The structural processing steps were as follows: the image noise was removed using a spatially adaptive non-local means filter and brain surface reconstruction; the individual functional space was spatially normalized to the MNI152 standard brain space; a customized group T1 template in the standard space was generated to reduce the error term that resulted from the image registration and bias in the template selection; and the fMRI images in the native space of each subject was registered to the standard space with a final resolution of 3 mm.

### Dynamic Functional Connectivity Network Construction

Because time-varying FC is complicated and differs from static FC, the recruitment of more regions in the associated networks could help to provide more precise information. A total of 142 regions that covered the cingulo-opercular network (CON), DMN, fronto-parietal network (FPN), occipital network (OCC) and sensorimotor network (SMN) as defined by Dosenbach et al. (2010) were selected. The cerebellum network was neglected because we sought to examine only the effects of aging on the higher-order brain network interactions and the dynamics of brain cognition and perception. Among these networks, OCC and SMN are involved in perception and primary visual and motion processing, respectively; the other three networks are important to higher-order cognitive functions. In each of these brain regions, time courses were extracted and averaged over a spherical region of interest (ROI) with a diameter of 6 mm. Then, a dynamic FC network was estimated using the sliding window Pearson correlation method, which yielded a series of 142 × 142 correlation matrices. We used a fixed-length rectangle window (width = 24 × TRs = 60 s), and the window was shifted by 1 TR. The obtained correlation series were then Fisher-Z transformed and low-pass filtered with a cut-off frequency of 1/w Hz. All of these network matrices were vectorized to simplify the analysis.

### Phase Randomization

As suggested previously (Hutchison et al., 2013a; Hindriks et al., 2016), phase randomization analysis was used to explore the dependability of the dynamic FC fluctuation. The processed rs-fMRI time courses from the senior and young groups were phase randomized into new time courses in which the frequency spectra of the bold signals were invariable. We called the phase processed data the null group, which was then compared with the senior and young groups. Phase randomization was conducted for all parameters except amplitude (Friston et al., 1994; Handwerker et al., 2012), which could preserve the temporal correlation properties. These steps were taken to allow for assessing the dependability of the dynamic fluctuations and to verify whether the FC fluctuations over the rs-fMRI involved specific neural activities.

### Functional Connectivity Variation (FCV)

The dynamic functional connectivity variation (FCV) was calculated as the standard variation of the dynamic FC series. In this approach, the stability of the FC fluctuation over time is quantitatively measured and compared between brain region pairs. Previous studies of resting-state fMRI have demonstrated that some intrinsic neural activities are related to the variations in FC. These findings likely suggest the internal mechanism of the resting-state fMRI; thus, the FCV matrix was calculated for each subject. The original FCV matrices and phase randomized FCV matrices of both groups were statistically analyzed and compared with the averages of each group using a one-sample *t*-test. Thus, we could easily examine network modularization and the FCV of each network or connection.

### Frequency Spectrum Analysis

A sliding rectangle window was used with a low-pass filtering effect on the functional fluctuations. The cut-off frequency was 1/*w* (1/60 Hz = 0.018 Hz). We assumed that the frequency spectrum of the dynamic FC fluctuation would change with aging; thus, we sought to specify the age-related changes in frequency or energy as age increased. The frequency band of the FC fluctuation was divided equally into two frequency bands, 0–1/2*w* Hz and 1/2*w*–1/*w* Hz, within which the fluctuation energies were calculated with a Fourier transform. Then, the energy ratio of the two frequency bands was calculated as the energy of the lower frequency band dividing the energy of the higher frequency band.

### Permutation Tests

To obtain robust results on aging-related variations within and between groups, a non-parametric permutation test with 5000 randomizations of the group labels was utilized for all measures described above. We defined the *t*-statistics between the two groups as the difference measurement, yielding a distribution of *t*-statistics after 5000 randomizations. Then, *p* = 0.001 was set as the threshold of significance. To better understand the results, both connectivity-based and network-averaged indices were examined. With the non-parametric approach, the size of the Type I error was guaranteed to be set at the prescribed significance level. The permutation test demonstrated an excellent ability to differentiate between different profiles, even when those profiles appeared to be highly similar.

### Sliding Window Length Analysis

Previous works (Hutchison et al., 2013a; Hindriks et al., 2016) have much discussed the influence of sliding window parameter settings. However, there have been no definite conclusions about the optimal window length. In this article, we also carefully assessed the influence of the window length on the variation and frequency spectrum of the dynamic FC. A sequence of window lengths from 2 to 256 time points was selected to examine the FC variation between the mean dynamic FC and the static FC. Another sequence of window lengths from 10 to 70 time points was selected to obtain the frequency characteristics of the dynamic function connectivity time series.

## Results

### Phase Randomized and Within-Group Analyses

**Figure 1:**
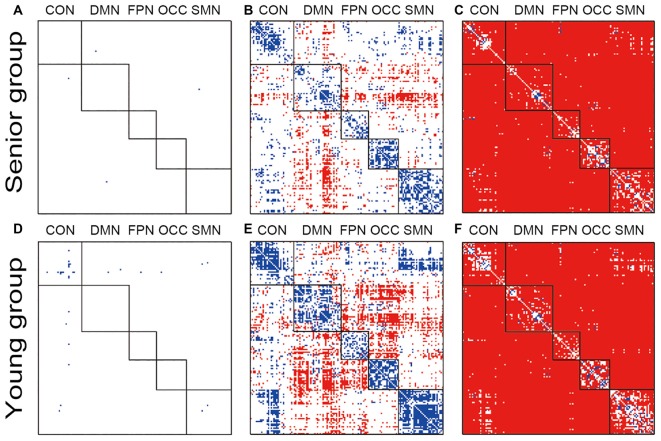
Illustration of the dynamic functional connectivity (FC) variation patterns within groups. Pictures **(A,D)** show the one-sample *t*-test results of senior and young null groups with phase randomization processed data (FDR *p*-value < 0.05; actual *p*-value < 0.0010); Pictures **(B,E)** show the one-sample *t*-test results of senior and young groups with the original data (FDR *p*-value < 0.05; actual *p*-value < 0.0011); Pictures **(C,F)** show two-sample *t*-test results between the original and phase randomized data within the senior and young groups (FDR *p*-value < 0.05; actual *p*-value < 0.0008). Red indicates a higher-than-average level in the one-sample *t*-test and a higher-than-null group in the two-sample *t*-test.

The within group and between group comparisons were conducted after passing the normality tests. Both groups lost the network organization pattern after phase randomization, and all of the within- and between-network connections showed similar FC variations (Figures 1A,D). From the original data obtained from both groups, clear modularization could be identified from the lower within-network variation and the higher between-network variation (Figures 1B,E). In the young group, higher variation of the dynamic FC was found in the DMN-related inter-network connections and the connections between the FPN and OCC. By contrast, in the senior group, only the DMN-related inter-network connections showed high variation. In both groups, however, the inter-network variations of the connections between the CON and SMN were clearly lower than the corresponding group averaged values; a similar result was obtained for the CON and OCC. Both the within- and between-network variations based on the original data were significantly higher than those of the phase randomized data for almost all of the connections (Figures 1C,F).

### Age-Related Changes in Static FC

**Figure 2:**
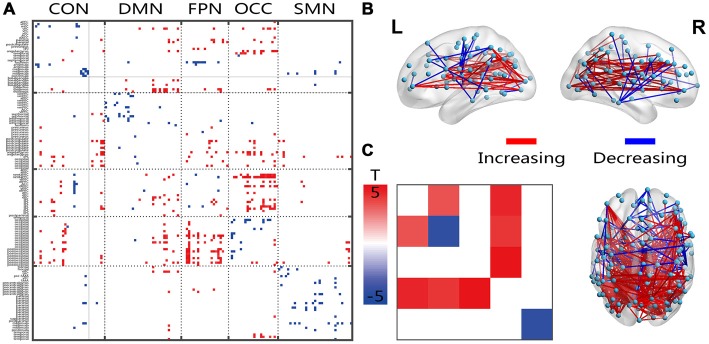
Illustration of the two-sample *t*-test results of the static FC compared between the senior and young groups. Pictures **(A,C)** depict the connections level and the network averaging level, respectively; Picture **(B)** is a 3D view of the results shown in picture **(A)** (constructed using the BrainNet Viewer). Red indicates an increase in the senior group; blue indicates a decrease in the senior group. The significant level is a *p*-value < 0.001.

Compared with the young group, all connections indicated decreased within-network FC in the senior group, and fewer within-network functional connections showed increases within the CON and OCC (Figure 2A). Most of the between-network connections, especially between the CON, DMN, FPN and OCC, showed increased FC in the senior group compared with those of the young group. From the 3D view in Figure 2B, more of the connections crossing the cerebral hemispheres were changed compared with the connections within one side. Most of the connections that both crossed hemispheres and occurred within a hemisphere were located between the posterior-anterior brain; in these connections, several distinct, intensively connected nodes were found. In the networks that were averaged and assessed, all or most of the between-network connectivities increased and the inner network functional connectivities decreased in the senior group compared with those of the young group (Figure 2C). However, only the DMN and SMN showed significantly decreased within-network FC.

### Age-Related Changes in FC Variation

**Figure 3:**
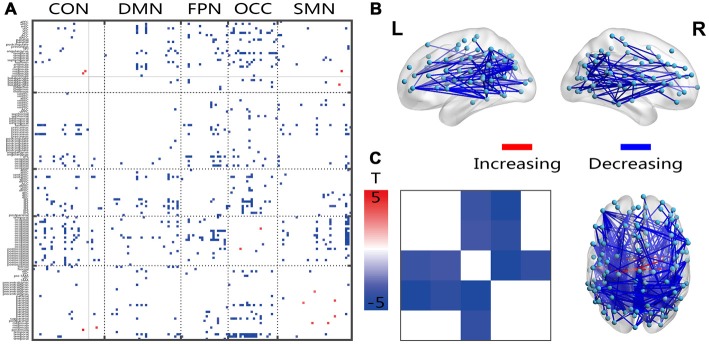
Illustration of the two-sample *t*-test results of the dynamic FC variation (FCV) between the senior and young groups. Pictures **(A,C)** depict the connections level and network averaging level, respectively; Picture **(B)** is a 3D view of the results shown in picture **(A)** (constructed using the BrainNet Viewer). Red indicates an increase in the senior group; blue indicates a decrease in the senior group. The significance level is a *p*-value < 0.001.

**Figure 4:**
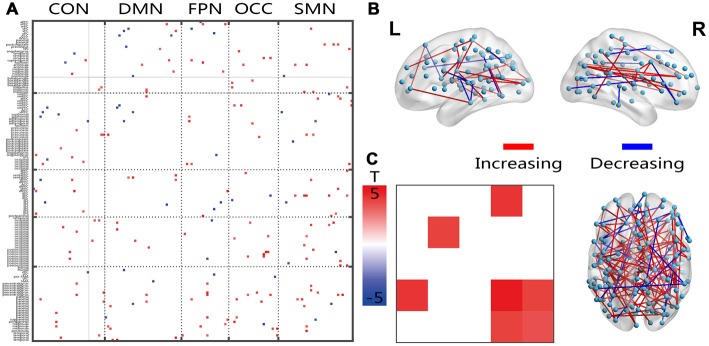
Illustration of the two-sample *t*-test results of the energy percentage in the low fluctuation frequency band compared between the senior and young groups. Pictures **(A,C)** depict the connections level and the network averaging level, respectively; Picture **(B)** is a 3D view of the results shown in picture **(A)** (constructed using the BrainNet Viewer). Red indicates an increase in the senior group; blue indicates a decrease in the senior group. The significance level is a *p*-value < 0.001.

Most of the significantly changed connections shown in Figure 3 were between-network connections. The few connections with increased connectivity were located between the hemispheres, and the changed connections shared a similar location with the FC connection, which crossed both hemispheres, showed a posterior-anterior distribution and was also changed (Figure 3B). Several intensively connected nodes were also obvious in the prefrontal and occipitotemporal regions. The averaging analysis of the networks showed that these decreases occurred only in the inter-networks, including all of the FPN-associated inter-networks, the OCC-DMN inter-network and the OCC-CON inter-network. The solid black lines in Figures 2–4 mark the subcortical regions.

### Age-Related Changes in Frequency Spectra

The connections that covered all networks showed increased energy ratios between the energies of the higher and lower frequency bands (Figure 4A). On average, the within-network connections, which included the DMN, OCC and SMN, and the inter-network connections CON-OCC and OCC-SMN indicated significantly increased ratios in the senior group. The 3D view revealed that these changed connections had both cross-hemisphere and anterior-posterior distributions (Figure 4B).

### Influence of the Sliding Window Length

**Figure 5:**
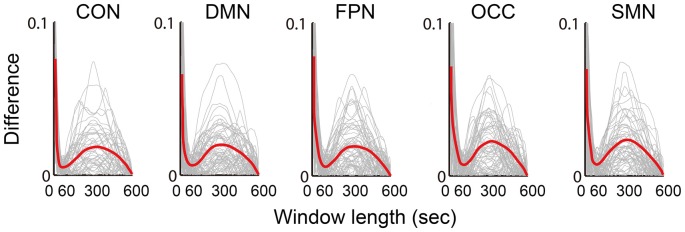
Illustration of the difference (absolute value of the difference) between the mean dynamic FC and the static FC. The results of five networks are individually averaged for all connections, which are plotted as gray curves; the red curves represent the means of all subjects from both groups.

The absolute difference between the mean dynamic FC and the static FC over the scan time was calculated and illustrated (Figure 5). Five network differences between the dynamic FC and the static FC are shown in the chart in Figure 5, in which the red central lines are the mean values and the gray lines indicate the values for different subjects. All five networks followed similar trends of variance, and when the window length was increased, the dynamic deviation of the static FC also varied. At approximately 50–60 s this difference reached a minimum, and at approximately 300 s the difference was at its maximum.

**Figure 6:**
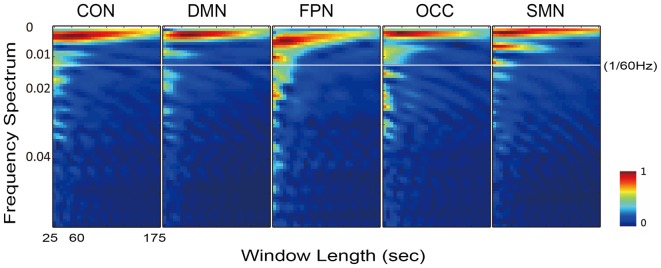
Illustration of the frequency spectrum, which varied with the sliding window length, for all five networks of one typical subject. The results of five networks were calculated from the individually averaged FC sequence of the within-network connections.

Accordingly, the frequency spectrums of the five networks from one typical subject also varied when the sliding window length increased from 10 to 70 time points (25–175 s Figure 6). The white line in Figure 6 is at 1/60 Hz and indicates the cut-off frequency of the low-pass filtering on the dynamic FC time series. With the increasing length of the sliding window, the energy of the higher frequency attenuated faster than the energy of the lower frequency. At frequencies above 1/60 Hz, the energy was very small, and at window lengths below approximately 60 s the energy difference was sufficiently stable for examination.

## Discussion

In this study, we showed the pattern of age-related changes in the dynamic connectivity profile between and within the whole-brain resting state networks. The originality of this study consisted of characterizing the age-related variation and frequency transition of whole-brain dynamic FC with the sliding window correlation approach. Several interesting findings were as follows: (1) in both groups, the FC variation showed distinct organization and age-related modularization, which were missing after phase randomization; (2) the FC variation indicated a significant decrease between networks, which increased with age and was dramatic in the FPN-associated and OCC-associated inter-networks; and (3) at a higher frequency, the dynamic FC showed an increased energy ratio in the senior group. These results not only shed light on the mechanisms of dynamic FC but also add to our understanding of normal brain aging. Here, we carefully discuss the results described above as well as the methodology of the sliding window correlation.

Similar to the functional specialization in FC, temporal variation of the connectivity also revealed a similar pattern or modularization, as illustrated in Figure 1. The whole-brain variations in the dynamic FC were not uniform across all systems and fell into two sides of the mean level within each group. The within-network connectivity showed a lower variation, and most of the between-network connectivity showed higher variation compared with the mean except for the CON-SMN and CON-OCC connections. Many previous studies have reported that the states of the dynamic FC matrices showed high variation in the between-network connectivity (Allen et al., 2014; Hansen et al., 2015). Allen et al. (2014) reported that seven reproducible states could be differentiated by connectivity between the DMN regions, indicating great variation. Those findings are consistent with the current results (Figures 1B,E), in which the inter-networks of the DMN showed high variation in both groups. No type of pattern among the networks was indicated in the phase randomized analysis (Figures 1A,D). This modularization of the FC variation was most likely associated with the intrinsic neural activities and interactions between the systems.

The pattern and modularization of the dynamic FC variation showed a clear decline in the senior group. The connections with higher and lower levels of variation both tended toward the mean, which indicated that less diversity in the variation between the subsystems occurred with increased age. A previous study (Du et al., 2016) reported that the FC of some connections showed less variation in schizophrenic patients. The changed modularizations of the FC variation in the two groups provided clear evidence that the modularization of the connectivity variation also declined with age, which also reflected functional integration and segmentation in aging brains (Hagmann et al., 2008; La Corte et al., 2016). Dedifferentiation of cognitive functions occurs in the aging brain, and many regions are reconfigurable to compensate for declines in other regions that occur with increased age (Sleimen-Malkoun et al., 2014). The dynamic variation in connectivity reflected that reconfiguration occurs all the time and follows some patterns, and when age increases, this pattern slowly changes. Previous studies have also reported that the brain regions dynamically participate or reconfigure into different modules during the scan time at rest (Bassett et al., 2011; Schaefer et al., 2014). The declining modularization of the FC variation in the senior group was likely related to aging and compensatory mechanisms.

The presence of FC variation, even within a single brain state (including the resting state), has been increasingly recognized (Chang and Glover, 2010; Hutchison et al., 2013a; Allen et al., 2014) and has been established as clinically relevant (Damaraju et al., 2014; Kucyi and Davis, 2014; Elton and Gao, 2015). A recent direct comparison of the awake, resting state with the anesthetized state has revealed a dramatic reduction in the connectivity variation during unconsciousness, which suggests that the connectivity variation is at least partly related to conscious operations (Barttfeld et al., 2015). One potential source of connectivity variation during consciousness is “mind wandering”, in which the brain consciously engages in different mental operations that produce fluctuations in the FC. However, we expected that the FC variation is a capacity of elasticity or operations to maintain states or connectivity transitions. This concept is similar to the cognition capacity resource, in which cognitive resources are limited and reduced in the aging brain. High variation of FC leads to the increased possibility of reconfiguration to address the loss of some resources. Qin et al. (2015) used the amplitude of the low frequency fluctuations (ALFF) of the dynamic FC to predict brain maturation between 7 years and 30 years of age. The findings of that study suggested that the increased variation was highly related to maturation. The ALLF was essentially the same with respect to the FC variation, both of which could be associated with the plasticity of the brain.

All decreased dynamic FC variations in the senior group were located in the between-network connections, especially in the FPN-linked and OCC-linked inter-networks. The inter-network between FPN and OCC was also indicated to be missing in the senior group (Figures 1B,E). These findings might indicate a posterior-anterior shift in aging (Davis et al., 2008; Vinette and Bray, 2015), which has been interpreted as compensatory in that higher-order cognitive processes are recruited to offset deficits in sensory processing. In all of the 3D views of changed connections, many posterior-anterior connections were also obvious. Cognitive and perceptual changes could be linked because they are susceptible to the same age-related factors, and a perceptual system decline could have an impact on the cognition outcome (Allen and Roberts, 2016). In young adults, visual learning engages an extended network of occipito-temporal, parietal and frontal regions, which is known to be involved in perceptual decisions (Kim and Shadlen, 1999; Shadlen and Newsome, 2001; Heekeren et al., 2004; Mayhew and Kourtzi, 2013). The subcortical regions, including the basal ganglia and thalamus, have been shown to be associated in cognition processing (Koziol and Budding, 2009). The results for the subcortical regions showed mainly increased connectivity in the DMN and decreased variation in the FPN and OCC. Considering the central or hub roles of both functional and structural networks, the subcortical regions might regulate passing signals or communication between the perception and cognitive systems, such as the OCC and FPN, the OCN and OCC, or the CON and SMN (Marchand et al., 2011). The increased fluctuation energy ratio of the high-frequency connected networks of the subcortical region might further reflect an age-related dynamic regulation, which requires further research in terms of behavioral and task fMRI experiments. With limited cognitive resources of the cognitive and perceptual systems in the aging brain, the interaction between these two systems declines as brain processing capacity is reduced.

Except for the declining capacity of cognitive resources, the changed FC fluctuation energy ratio of the higher frequencies could allow for a deeper inspection of the dynamic communication between regions. Shakil et al. (2015) found that the dynamic FC has a frequency dependance. Fluctuations within the frequency of 0–1/w were also suggested, which implied a real, physiologically dynamic connectivity (Leonardi and Van De Ville, 2015). The connectivity fluctuation is a type of intrinsic interaction of different frequency components involved in different neural activities. The speed of processing in the aging brain (Park and McDonough, 2013) is also an important property that reflects cognitive resources. Aging-related decreases in the amplitudes of low-frequency BOLD signal fluctuations have been observed, suggesting that the low-frequency fluctuations of neuroactivities are more vulnerable to aging-associated declines (Hu et al., 2014). We thought that the increased energy ratio of the fluctuating high frequencies was most likely caused by increased damage to the fluctuations of the low frequencies. The decreased variation and increased speed of fluctuations in the dynamic FC would result in disorder of the dynamic communications between different brain regions in senior individuals.

Logically, in the sliding window correlation method, the window length should have a substantial effect on the captured connectivity fluctuation. This factor was the most important consideration in terms of the overall accuracy of the technique (Leonardi and Van De Ville, 2015; Hindriks et al., 2016; Shakil et al., 2016). However, there is still no clearly determined standard for window length selection. Convergent results suggested that a window length between 50 s and 60 s is optimal. As we expected, the dynamic FC during rest was found to be a dynamic balance that maintained intrinsic connectivity patterns and even vigilance for cognitive tasks. Moreover, the variation in the FC was predicted to be centered around the static connectivity level. This expectation was supported by the appearance of a minimum on the difference curve between the mean dynamic and the static FC, as shown in Figure 5. The maximum was located at approximately 300 s (5 min), which was consistent with the suggestion of a longer scan time for reliably detecting resting state connectivity (Heekeren et al., 2004; Zuo et al., 2013). The individual frequency spectrum changed with the window length, as shown in Figure 6; this result indicated that a window shorter than 60 s retained potentially important energy for all of the networks, and the lower frequency energy contributed most of the connectivity fluctuation. A previous study (Leonardi and Van De Ville, 2015) suggested a meaningful frequency of under 1/w, which was even lower than the commonly considered frequencies for BOLD fluctuations. A fluctuation frequency of 0–1/w was suggested previously (Leonardi and Van De Ville, 2015) and employed in a later study (Qin et al., 2015; Liu et al., 2016). In the present article, the frequency was even lower than the commonly considered 0.01–0.10 Hz of the BOLD signal fluctuation, which was in accordance with the neural activity. This type of mismatch could be understood given that unlike BOLD signal fluctuations, which indicate direct neural metabolic activity, the fluctuation in the functional connectivities reflected interactions between the different regions. The interactions were also revealed in the current results, which showed that more of the affected connections were located in inter-network and inter-hemispheric connections. Furthermore, we believe that the FC variation must depended more on the fluctuation frequency, which is influenced by various factors, including anatomy, cognition, physiology and disease. The more that we understand the physiology of dynamic FC and the methodologies used to study these phenomena, the more insights we will have into the mechanisms of aging and disease.

## Conclusion

In conclusion, this study presented a resting-stated dynamic FC analysis of normal brain aging. All of the results converged to expound compensation and reorganization of the networks during aging. We examined the modularization of the FC variations in the brain and decreased modularization in the aging brain. Additionally, decreased variation and increased damage to the low frequency fluctuations of the dynamic FC with aging were detected; these changes were interpreted to be associated with declining cognitive resources and limited processing speeds in the senior brain. In this article, we provided and applied new insights into FC analysis for use in aging research. The results indicated that the dynamic features of the resting-state FC were actually the intrinsic interactions between regions and cognitive resources. When some cognitive resources were reduced in aging, this type of dynamic mechanism acts to reconfigure or even train a new cognitive resource. Our conclusions in this article were fully supported by dependable results; we believe that the dynamic FC can potentially capture the intrinsic rules of compensatory processes in the aging brain, and that the present results will promote insightful understanding of spontaneous fluctuations in FC as well as aging mechanisms.

## Author Contributions

MS, YL and XiongZ processed all the image data and conducted some analysis work; YC and WW were in charge of the analysis work and wrote the manuscript; JM and HN provide some useful guidance and ideas; YC, XinZ and DM designed and provided the original idea; XinZ and DM sponsored the whole research.

## Conflict of Interest Statement

The authors declare that the research was conducted in the absence of any commercial or financial relationships that could be construed as a potential conflict of interest.

## Footnotes

^1^http://fcon_1000.projects.nitrc.org/indi/pro/nki.html

^2^http://lfcd.psych.ac.cn/ccs.html
